# Population parameters of *Drosophila* larval cooperative foraging

**DOI:** 10.1007/s00359-024-01701-w

**Published:** 2024-04-10

**Authors:** Amy Liao, Christy Qian, Sepideh Abdi, Peyton Yee, Sean Michael Cursain, Niav Condron, Barry Condron

**Affiliations:** Department of Biology, University of Virginia, Charlottesville, VA 22901 USA

**Keywords:** Drosophila, Foraging, Cooperation, Social behavior, Group membership, Fitness

## Abstract

Cooperative foraging behavior can be advantageous when there is a common exploitable resource. By cooperating, members of the group can take advantage of the potential of increased efficiency of working together as well as equitable distribution of the product. An experimental signature of cooperative foraging is an Allee effect where at a certain number of individuals, there is a peak of fitness. What happens when there are intruders especially ones that do not contribute to any work required for foraging? *Drosophila* larvae secrete digestive enzymes and exodigest food. Under crowded conditions in liquid food these larvae form synchronized feeding clusters which provides a fitness benefit. A key for this synchronized feeding behavior is the visually guided alignment between adjacent larvae in a feeding cluster. Larvae who do not align their movements are excluded from the groups and subsequently lose the benefit. This may be a way of editing the group to include only known members. To test the model, the fitness benefit from cooperative behavior was further investigated to establish an Allee effect for a number of strains including those who cannot exodigest or cluster. In a standard lab vial, about 40 larvae is the optimal number for fitness. Combinations of these larvae were also examined. The expectation was that larvae who do not contribute to exodigestion are obligate cheaters and would be expelled. Indeed, obligate cheaters gain greatly from the hosts but paradoxically, so do the hosts. Clusters that include cheaters are more stable. Therefore, clustering and the benefits from it are dependent on more than just the contribution to exodigestion. This experimental system should provide a rich future model to understand the metrics of cooperative behavior.

## Introduction

Cooperative behavior emerges amongst many animal taxa as a way to more efficiently forage for a common resource (Allee 1927; Vickery et al. 1991; Nowak 2006; Raihani et al. 2012). A key feature of animal aggregations is a potential for intra-specific competition for resources. It follows that there should be a decrease in fitness with increasing animal density. This would be offset by the benefits of aggregation so that there should be an optimum: enough members to have useful cooperation and yet not too many to excessively deplete resources. This yields an “Allee effect” or the optimal number or density at which cooperation is maximally beneficial (Allee 1927). This is a very useful number as it points to the parameter space where the evolution of cooperation is likely operating and is critical experimentally for model systems of this behavior. However, a common resource can also be subjected to exploitation, either from within the group by cheating (Lindstedt et al. 2018) or other species by raiding. Because of their small pharynxes, fly larvae are thought to use their large salivary glands to secrete, digest and breakup food in their external environment before ingestion (Gregg et al. 1990; Scanvion et al. 2018; Beyramysoltan et al. 2020), creating a common resource. This is likely a rich location for various aspects of social behavior, including cooperation, cheating, raiding and mutualism.

*Drosophila melanogaster* larvae in liquid and at high density form cooperative feeding groups called clusters (Dombrovski et al. 2017). These clusters require social learning (Dombrovski et al. 2019) and provide a fitness benefit to the resultant adults (Dombrovski et al. 2020). The key feature of these clusters is the synchronized digestion and mixing of external food by all larvae. In such an arrangement, secreted digestive enzymes and ingested food are likely mixed and shared communally. However, larval cooperation requires visually-guided synchronization of large movements (Dombrovski et al. 2017). This involves a critical period of visual plasticity in which animals learn to match movements to neighbors. Animals who do not go through this critical period do not spend much time in clusters and produce smaller adults (Dombrovski et al. 2020). Therefore, this critical period might act as a filter, or password generator, to seal a cluster with a coordinated locomotion cycle to keep interlopers out. This provides an excellent experiment model for a deeper neuroethological understanding of cooperation. This is especially true for fly larvae, which have an array of tools to manipulate circuits and the availability of a complete synaptic connectome (Winding et al. 2023).

In order to develop the larval cluster experimental model further, a set of Allee measurements were made for 4 lab strains. Two extra strains, one blind, and one with depleted salivary glands were also tested alone and in mixes. The salivary gland-depleted strain is an obligate cheater with respect to contributions to a common exodigestion mix. This allows a deeper investigation of the values of how larvae with potentially different clustering contributions might cooperate or not.

## Materials and methods

### Fly stocks

**Table:** 

Designation	Description	Species	Notes
CS	CantonS	D.melanogaster	Ed Lewis, Caltech
P	White-eyed host	D.melanogaster	Bloomington #24,055
GMRhid	Blind	D.melanogaster	Bloomington #5771
sgs	sgsgal4	D.melanogaster	Bloomington #6870
hid	UAShid	D.melanogaster	Bloomington #65,403
tai	UAStai	D.melanogaster	Bloomington #6378

### Fly stock maintenance and egg collection

All Drosophila melanogaster strains were raised in standard Caltech food vials containing (1000 ml molasses, 14000ml H_2_O, 148 g agar, 1000 ml corn meal, 412 g Baker’s Yeast, 225 ml Tegosept, 80 ml propionic acid). Pre-processed vials were prepared as described (Dombrovski et al. 2020). Video analysis was as described (Dombrovski et al. 2017). Larvae were kept at 24 °C, 30% humidity. For egg production, ~ 50 adult flies 3–4 days old were transferred into egg cups and kept in the same conditions. Eggs were always collected on 35 mm petri dishes containing standard agar-molasses food and yeast.

### Cluster measurement

2D assays were then made with third instar clustering larvae from the vials as described (Dombrovski et al. 2017). 40 larvae were added to a 2D apparatus with pre-pre-processed food and video recorded. The proportion of larvae clustering at 240, 360 and 480 min in clusters was recorded and averaged. The average of these assays was used to establish the clustering rate. These time points were chosen so as to be consistent with previous studies (Dombrovski et al. 2017, 2019, 2020; Williamson et al. 2021).

### Wing size measurements

40 s instar larvae were added to a pre-processed vial and incubated to adulthood. Wing size of frozen females was measured using a described technique (Stuart Gilchrist and Partridge 1999) (distance from the base of the alula to the distal end of the third longitudinal vein). A single wing from each animal was removed and mounted on a slide along with more than 10 from a single vial, with at least 3 vials per genotype/condition. High-quality images of the slide were taken with a camera mounted on a tripod for subsequent wing size assessment using ImageJ (see below). Values were then averaged to give an estimate of the wing size for a designated genotype/condition.

### Photography and video recordings

For cluster frequency analysis, videos were recorded on an iPhone 5 at full resolution and 1 frame/60” using “Lapseit” software for iOS. For wing images, an iPhone 10 was used. Video analysis was further performed in iMovie and ImageJ (32-bit version for Windows).

### Statistical analysis

Unless otherwise stated, all data are presented as mean values and error bars represent standard deviation or stand error as indicated. Statistical significance was calculated by one-way ANOVA using Tukey’s method. All data was tested for normalcy before using Tukey’s method. *p < 0.05; **p < 0.01; ***p < 0.001. Analysis was conducted using the GraphPad Prism 8 statistical software.

## Results

**Fig. 1:**
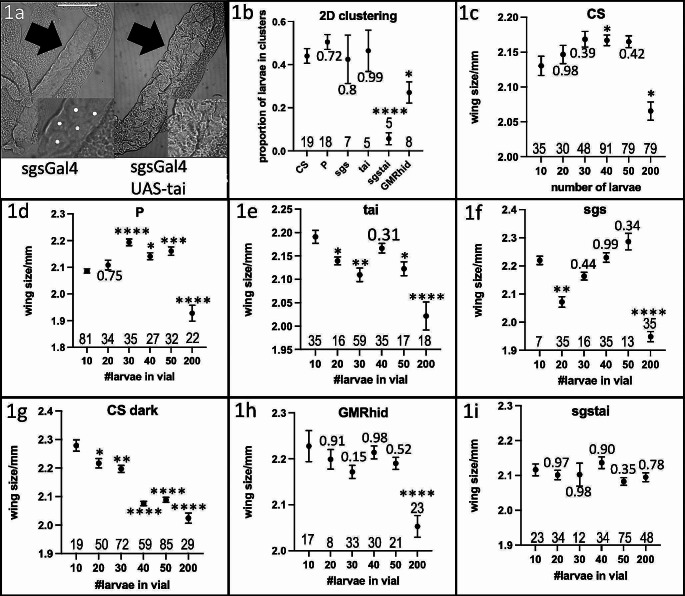
Allee analysis of fitness in lab strains. (**a**). Salivary glands of sgs (sgsGAL4) and sgstai (sgsGAL4/UAStai) in the first 24 h of the 3rd instar. Salivary gland cells are visible in sgs but not in the sgstai samples. Sale bar is 200 μm. (**b**). 40 larvae from the 6 strains used in this study were placed in a 2D clustering assay. The average number of larvae in clusters from each assay was averaged over several experiments and indicated. About half of the larvae are in clusters for the 4 normal strains but greatly reduced in blind larvae and almost absent in cheater sgstai. Values are expressed as the average of multiple assays and the standard error of the mean. The number of samples is shown for each data point. Significance, after normality test, is based on comparison to CS and ANOVA analysis followed by Tukey’s Test. * *p* < 0.05; **** *p* < 0.0001. Probabilities for non-significant differences are shown. (**c-i**). Various numbers of L2 larvae were added to pre-processed vials and raised to adults. The size of the female wing was used as a fitness measure. For CS, tai, sgs, P strains there is a peak or plateau point in wing size at about 40 larvae per vial. Placing CS in the dark, where clustering is inefficient results in a steady decay curve likely due to competition. Indicated are the averages and standard deviation as errors. The number of samples is shown for each data point. Significance, after normality testing, is based on comparison to wing size at 10 animals and ANOVA analysis followed by Tukey’s Test. * *p* < 0.05; ** *p* < 0.01; *** *p* < 0.001. **** *p* < 0.0001. Probabilities for non-significant differences are shown

Cooperative foraging behavior is thought to require social exodigestion of food, and therefore likely requires functional salivary glands. To test the role of salivary glands, clustering assays were performed (Dombrovski et al. 2017). One wild-type strain (CS) and three transgenic lab strains (P, sgs, tai, see methods) were used. In addition, a blind GMRhid and the cross of sgs and tai, sgstai (see methods) were also tested. Blind GMRhid larvae are very inefficient at clustering due the requirement of vision for this synchronized behavior (Dombrovski et al. 2019). The sgstai larvae are expected to have non-functional salivary glands due to the overexpression of the metamorphosis-inducing gene *taiman*, tai (Farkaš et al. 2014). In comparison to wild type, sgstai larvae have reduced salivary glands (Fig. 1a). Initially, the apoptosis-inducing gene hid (see methods) was overexpressed in salivary glands but this proved mostly lethal at the pupal stages. Salivary glands produce a glue which allows pupae to stick to the vial sides, an event which did not generally occur in sgsGal4/UAS-hid. Therefore sgsGal4/UAS-tai (sgstai) was used as a salivary gland depleted animal. Each of the 6 strains were first tested for clustering in a 2D assay (Dombrovski et al. 2017). Pre-processed food was placed between two glass slides, and the 40 clustering larvae from a crowded vial were loaded and video documented for 24 hours. Clustering was measured as the proportion of larvae in clusters as described (Dombrovski et al. 2017). Clusters were defined at 4 or more synchronized larvae in the food. The clustering proportion was averaged from 240’, 360, and 480’. About 50% of larvae were in clusters for the wild type and 3 lab transgenic strains (Fig. 1b). Few of salivary gland depleted sgstai clustered while loss of vision (GMRhid) halved clustering rates as described before (Dombrovski et al. 2019). The sgs and tai are the parental strains for sgstai and this shows that salivary glands are likely a key part of clustering.

**Fig. 2:**
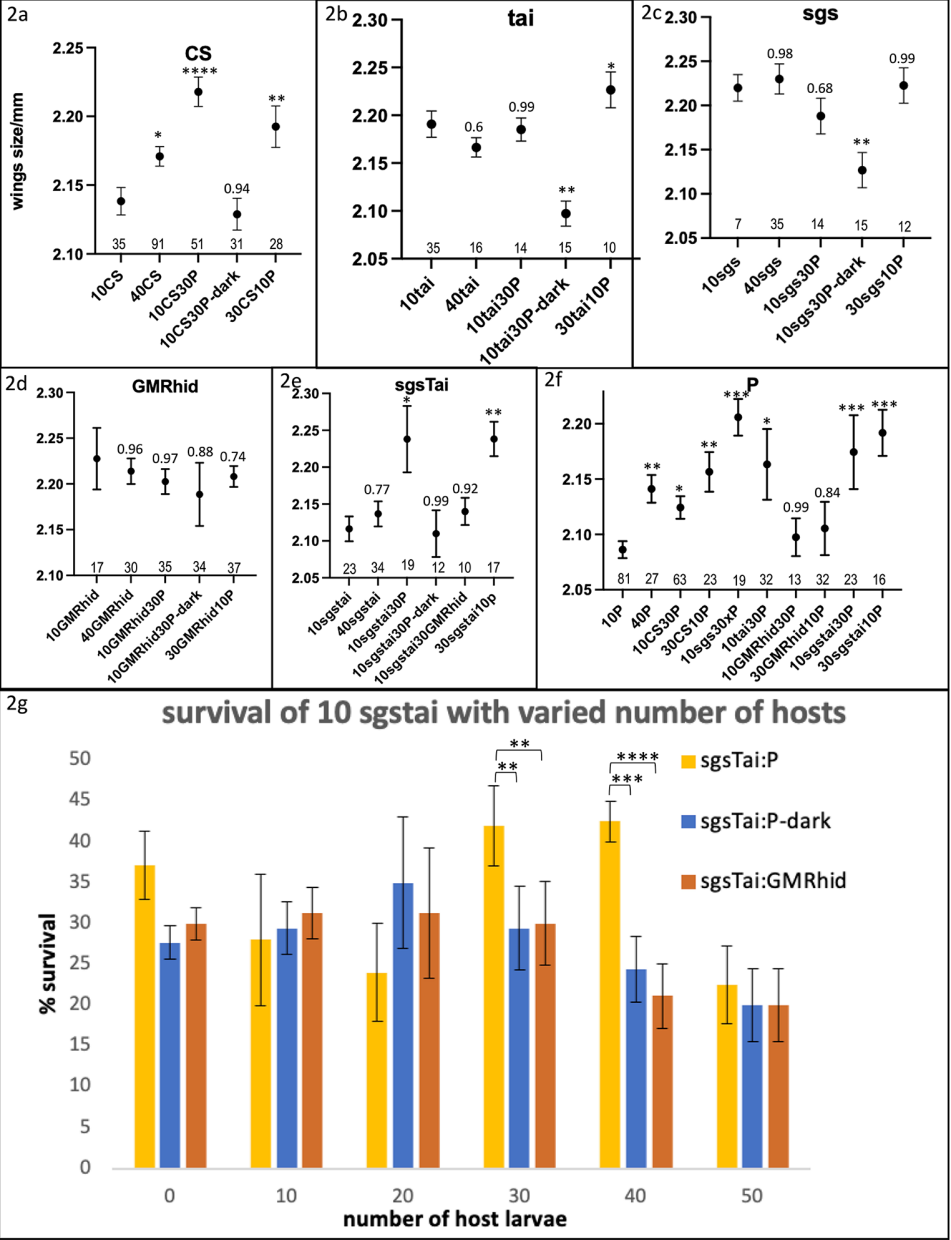
Fitness of larval mixtures. (**a-f**) Wing size fitness of 40x larvae/vial grown to adult of select mixes for 6 labs strains including blind GMRhid and salivary-gland-depleted sgstai. Significance, after normality testing, is based on comparison to wing size at 10 animals and ANOVA analysis followed by Tukey’s Test. * *p* < 0.05; ** *p* < 0.01; *** *p* < 0.001. **** *p* < 0.0001. Probabilities for non-significant differences are shown. Values are expressed as the average of the indicated number of samples and the standard deviation. (**a**). CS: except for development in the dark, all 40x larval combinations with various amounts of the P strain show gains over 10x. (**b-c**). tai/sgs: These strains have no distinct Allee peak but fail to show decreased fitness as numbers grow until about 40. This gain is lost in cluster free dark-developed vials. P can substitute for either sgs or tai. (**d**). GMRhid (blind): No gain or loss is seen for 10-40x larvae of any mix. (**e**). sgstai (larval gland depleted): Gain is seen for sgstai when mixed with P either 10:30 or 30:10. This is lost when P is replaced with blind GMRhid or incubation in dark. (**f**). P (host strain): P strain shows a gain from 10 to 40 larvae and P can be substituted for CS, sgs, tai, sgstai but not blind GMRhid. Both P and sgstai (e) gain from mixing. (**g**). Survival of various sgstai mixes with P, P-dark or GMRhid compared at increasing larval density. In each case, 10 sgstai were placed in a vial in increasing numbers of hosts as indicated on the X-axis. At 30 and 40 P, there is greater survival of sgstai that with GMRhid as hosts or P in the dark. Significance, after normality testing, is based on comparisons between dark reared and use of blind GMRhid as host and calculated with ANOVA analysis followed by Tukey’s Test. ** *p* < 0.01; *** *p* < 0.001. **** *p* < 0.0001. Values are expressed as the average of the indicated number of samples and the standard deviation

Cooperative foraging behavior should also exhibit an Allee effect in terms of the number of larvae in a defined substrate. There is expected to be an optimal number of cooperating larvae in a vial in which the positive effects of the group offsets competition. Previous studies used 200 larvae per vial (Dombrovski et al. 2017, 2020) which also likely featured significant intra-specific crowding effects (Miller and Thomas 1958; Venkitachalam et al. 2022). In order to find the optimum, a range of different numbers of second instar larvae were added to preprocessed vials as described (Dombrovski et al. 2017). Preprocessed vials have previously hosted about 100 wild type CS larvae from egg to pupal stages and about half of the about 5 ml of food was now in a semi-liquid, partially digested state (Dombrovski et al. 2017). The rationale for using these vials is that food is optimized for clustering based on physical properties, digested state and microbiome. Therefore, larvae entering these vials should be able to cluster. Larvae were loaded at the second instar stage so that they could enter the critical clustering period at the start of the third instar (Dombrovski et al. 2019). Vials were loaded with 10, 20, 30, 40, 50, or 200 larvae. The expectation is that any measure of fitness should decay with an increasing numbers of larvae due to competition but there should also be a peak in the distribution due to cooperation (Allee 1927). Larvae were allowed to develop into adults and female wing size was assessed as a fitness marker. Female wing size is directly related to body size and scales with fecundity (Gilchrist and Partridge 1999, 2001). Wing size is also directly related to how much larval clustering occurs (Dombrovski et al. 2020; Williamson et al. 2021). CS and P (Fig. 1c, d statistics Fig. 2a, f) show a fitness peak of about 40 larvae per vial. The fitness gain for 40 P larvae, over 10, remains consistent for mixes with CS, sgs, tai, sgstai but not for mixes with blind GMRhid or with any combinations in the dark (Fig. 2f). The lack of difference for fitness between 10x and 40x larvae for sgs and tai might be due to greater intraspecific competition. But for measuring optimal clustering conditions for CS and likely other strains, about 40 larvae per vial is optimal.

If 40 larvae is the optimal number in a vial for the fitness benefits of clustering, can these larvae be of mixed composition? If so, it is predicted that sgstai larvae should be able to gain from a wild type host. The P strain, which has distinguishable white eyes, was used as a host for all other strains. A total of 40 L2 larvae were loaded into each vial, grown to adulthood and ha their wings measured. Either 10x test and 30x P or 30x test and 10x P were used. CS and P are interchangeable with each other (Fig. 2). 30xP substitutes for 30xCS (Fig. 2a) and vice versa (Fig. 2b). This is also true for the sgs and tai strains (Fig. 2c-d). However, this effect is lost when the vials are kept in darkness which attenuates cooperation (Fig. 2a-d). Therefore, the Allee effect gain in going from 10 to 40 larvae does not matter on composition. However, this is not true for blind GMRhid larvae. Substituting 10xP with 10xGMRhid along with another 30xP removes the Allee effect gain. Blind larvae presumably block clustering in the host by interfering with group synchronization (Dombrovski et al. 2020). sgstai larvae, which do not cluster well, and do not show an Allee effect by themselves, now show a gain in wing size when mixed with P. This is presumably due to the salivary gland-depleted strain using the salivary products of the host. However, this occurs with both 10:30 and 30:10 proportions. The expectation had been that going from 10:30 to 30:10 cheaters to host, that the gain would not be seen. In fact, the depleting effect of excess sgstai ‘cheating’ was expected to be seen in the P hosts, which should have lost their Allee effect. Instead, they show gain (Fig. 2f). Therefore, salivary gland depleted larvae show gain with a wildtype host which also gains from the mix. This gain can also be seen in adult survival (Fig. 2g). Survival decreases with increasing numbers of larvae in the vial but there is an increase in wing size at 10sgstai and 30P compared to the same blend in the dark or using GMRhid as host (Fig. 2g). Therefore, the increase in wing size matches survival.

**Fig. 3:**
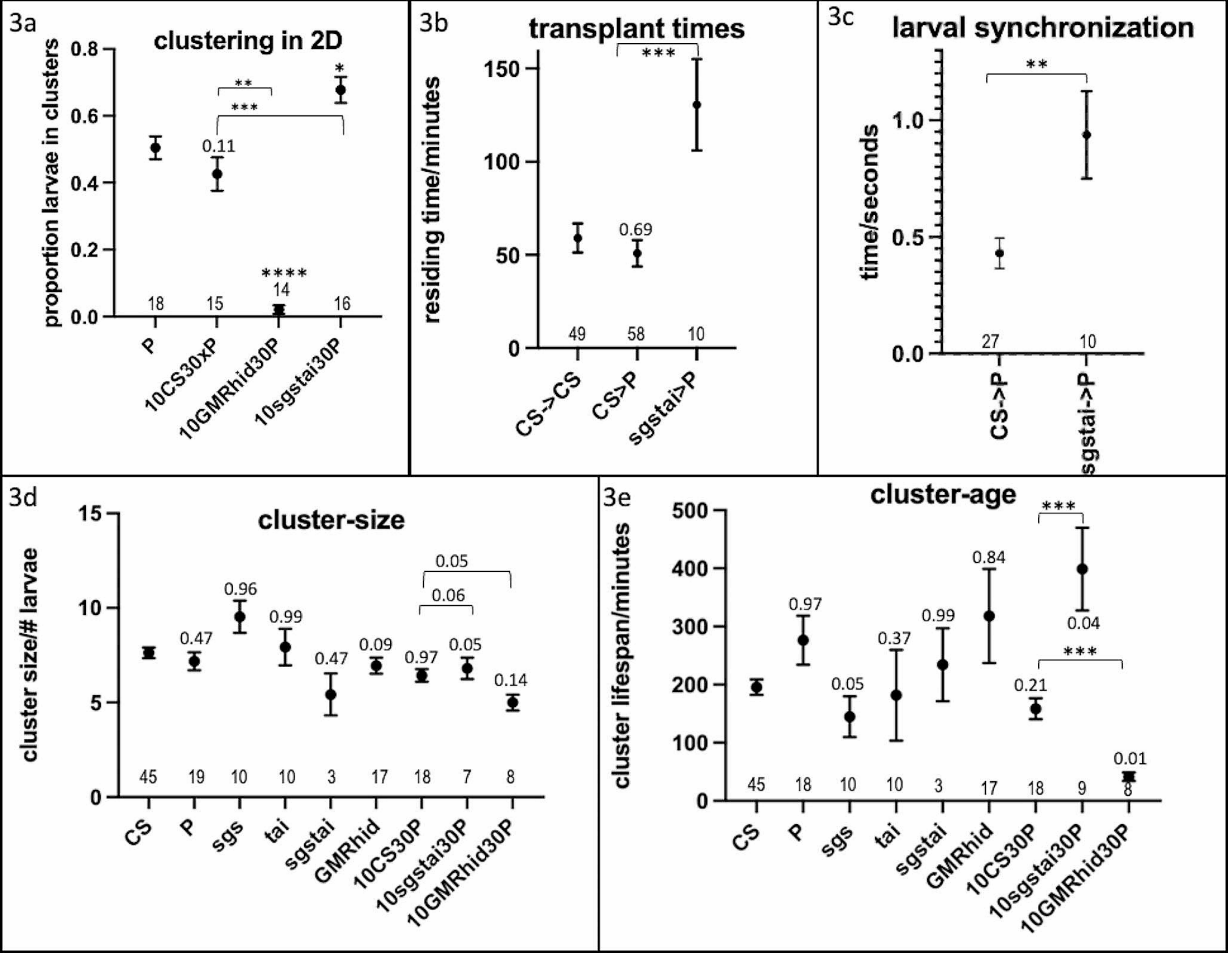
Clustering dynamics of mixes. (**a**). The proportion of larvae clustering in 2D was measured for three key mixes: P alone, 10CS&30P, 10GMRhid&30P, and 10sgstai&30P. About half of larvae are in clusters for P, 10CS&30P. This is about the same for CS alone (Fig. 1a). GMRhid blocks all clustering in P. However, sgstai increases the amount of clustering when substituted for P or CS. All graphs in this figure show the average with standard error. The number of samples is shown for each data point. Significance was calculated, after a normality test, by ANOVA analysis followed by Tukey’s Test and compares each sample to the first one on the left side of the graph. Other comparisons are as indicated. * p < 0.05; ** p < 0.01; *** p < 0.001; **** p < 0.0001. P values for samples without significance are indicated. (**b**) Clustering larvae were removed, food color labeled and transplanted back and monitored. CS larvae spend about an hour in CS or P clusters. sgstai larvae spend about twice this time. (**c**) Larvae synchronization of CS into P or sgstai into P transplants. CS synchronize their movements by about 0.5 seconds, out of a 2-second locomotion cycle. This is about the same as CS into CS. sgstai synchronize less and are close to that of blind or naïve larvae. (**d**) Clusters in various strains and mixes were monitored for their average size. The number of larvae in each cluster is about 7 animals. Blind GMRhid larvae have reduced clustering but their clusters are about the same size. Only 3 sgstai clusters were seen and these have about the same size as other clusters. (**e**) Clusters in 3d were monitored for their average lifespan. All are about 3 hrs with the exception of 10sgstai30P and 10GMRhid30P. Blind GMRhid reduces host cluster lifespan 4 fold from 156’ to an average of 41’. sgstai raises the life span about 2.5 fold to 298’

To investigate the mutual gain between P and sgstai further, 2D clustering assays were performed with blends. 10CS&30P and P cluster at about the same amount while more larvae cluster in 10sgstai&30P. GMRhid block all clustering when mixed with 30P (Fig. 3a). Therefore, sgstai increases clustering when mixed with a wild type host. To examine inclusion, food coloring-labeled larvae were add to either CS or P clusters and the length of time spent in the cluster measured (Dombrovski et al. 2017). Transplanted CS larvae spend about an hour in either CS or P clusters as measured before (Fig. 3b). sgstai larvae spend over double this time in P clusters (Fig. 3b). To examine if this increased cluster inclusion time is based on better synchronization (Dombrovski et al. 2017), high-resolution videos were made of transplanted CS or sgstai larvae. The sgstai larvae do not synchronize with host (Fig. 3c). In previous studies, such non-synchronizing larvae did not stay long in clusters (Dombrovski et al. 2017, 2019; Williamson et al. 2021). Blind GMRhid larvae delay inter-larval movements at 0.72s+/0.06 (Dombrovski et al. 2017)which is similar to that between sgstai and P (0.72s+/-0.06) and much longer than between CS and P (0.43+/-0.06 s) reported here. To examine the clustering dynamics, 2D assays were established and every cluster within the first 6 h documented. The number of larvae in each cluster was counted and followed for its duration. The cluster size was the average number of larvae for the lifespan of the cluster. This was about 7 for all conditions except sgstai and 10GMRhid&30P. Only 3 clusters were seen for sgstai and those for 10GMRhid&30P were close to the limit for the definition of cluster, which is 4 larvae. The average size of sgstai mixed clusters was about the same as that of P and so this does not explain the increased clustering. The average cluster life span was also measured. This was larger than 10CS30P. Therefore, sgstai larvae increase overall clustering by increasing the lifespan but not the size of a cluster (Fig. 3e).

## Discussion

Cooperative foraging should confer an Allee effect in fitness, where at an intermediate density of individuals, there is an advantage to individuals that offsets the intra-specific competition. For fly larvae, this is about 40 animals per vial. With more larvae, there is increased competition for fewer resources and cooperative clustering is not efficient. Obligate cheating larvae do not cluster but do so avidly with normal hosts. This confers an increase in fitness to both the cheaters and the hosts. This is likely via increased cluster stability.

The parameters of clustering estimated in this study need to be interpreted only in the context of the experimental setup. The food, secreted digestive enzyme content, microbiome and the 2 dimensionality of the clustering apparatus very likely influence the measured parameters and such conditions might be rare in the wild. For instance, the food might be rich enough that digestive cheating might not be needed, and the 2 dimensionality might force cooperation while in a natural environment this might not occur. Many examples exist of features of *Drosophila* laboratory physiology and behavior which might not represent what is commonly seen in the wild (Markow 2015). Nevertheless, the methods and parameters used here allow for more rapid dissection of the neural circuits that elicit this complex behavior. Once these circuits are understood, it will be necessary to relate their function to more realistic conditions.

In the absence of clustering, (Fig. 1, blind GMRhid and CS-dark), there is a steady decay in fitness as the number of larvae increase, likely due to a steady increase in internal competition. However, for the 4 standard strains, there is a shoulder on this curve at about 40 larvae/vial which likely represents an Allee effect. At just the right density, clustering makes sense in the context of adult wing size. While clustering might have many other selective advantages in the wild, each with different parameters, in a controlled lab environment, it is 40 larvae. An odd observation of this data is that there is no disadvantage to cheater sgstai at high densities (Fig. 1, sgstai, 200 larvae). For all tested larvae, including in the dark and blind animals, there are signs of loss of fitness at the highest density, 200 larvae per vial. This is not true for sgstai. These larvae show no density-dependent loss of fitness. Given that they are lacking salivary function and likely depend on microbiomic support for exodigestion, it might be that it is salivary products that create the competition. Excess exodigestion might remove some critical intermediates. There might also be salivary products that may be toxic to larvae. There are known toxic compounds at high larval density (Belloni et al. 2018) and maybe some of these are direct products of salivary glands.

A striking result of the cluster-mixing experiment in Fig. 2, is that the 4 lab strains are interchangeable for fitness. These vials are loaded with second instar larvae and assuming that all go through the L3F1 visual critical period (Dombrovski et al. 2019), they should be able to learn to cluster together. This means that there are not overt barriers to cluster mixing. It will be important in future experiments to see if wild caught strains mix productively, especially those of different allied species. Data from carrion-eating larvae indicate that species mixing is common (Charabidze and Aubernon 2023). On a carcass or a rotting piece of fruit, it might make more sense for all exodigesting larvae to cooperate rather than compete. This might be an environment very prone to cheating or raiding in various ways. Given that salivary gland depleted larvae can incorporate into clusters and increase their own and the host fitness, the value of clustering in this model must be more complex than simple addition of digestive enzymes. Indeed, the clustering fitness is not dependent on the number of cheaters to host ratio. This implies that the contribution of salivary glands to fitness exists, as cheaters need hosts, however not much product is needed. It will be critical to investigate the exact nature of the nutrition that is occurring in clusters. Knowing the specific nutrients might point to the values sought after in clustering.

Previous studies indicate that cluster membership is directly related to adult fitness. (Dombrovski et al. 2020; Williamson et al. 2021). This is largely substantiated in this study. Cheater sgstai increase their own and host fitness by increasing cluster stability. So more clustering gives bigger adults. However, previous studies had also indicated that cluster residing time was related to how well a larva synchronized with its neighbors (Dombrovski et al. 2017, 2019, 2020; Williamson et al. 2021). sgstai larvae do not synchronize well with their neighbors and yet stay longer than host in a cluster. They likely do not cluster well as they never learned it during the early third instar critical period because little or no clustering happens for this strain. However, they can nevertheless stay in clusters even though not synchronizing. This could be that they are using means other than synchronized locomotion to keep aligned with their neighbors, or perhaps using some other mechanical scheme like gripping other larvae. This also means that the stability of a cluster is dependent not only on synchronization but also on individual residing time. The average lab strains spend just under an hour in a cluster and those clusters generally survive about 3 h. Individual sgstai larvae spend over two hours in a cluster and those clusters last over 6 h. It is, perhaps, the higher turnover in normal lab strain clusters that shortens their lifespan. This raises the question as to why larvae don’t stay longer in clusters? No doubt clusters occurring in the wild face far more challenges than in the lab and a shorter lifespan might be advantageous.

This study raises an important consideration for the many published larval density experiments. It is critical to understand the social behavior of the larvae before considering the fitness outcome. The fitness benefits or losses need to be considered in light of local aggregation and clustering. Here clustering has a strong effect, because it maximizes animal proximity. Aggregation, before clustering, is another phenomenon that has yet to be studied in the context of these fitness experiments (Mast et al. 2014). Finally, it can be concluded that clustering is a far more complex behavior than previously described but is a powerful experimental system for which to study the neuroethology of social behavior.

## Data Availability

No datasets were generated or analysed during the current study.
